# Skeletogenic Fate of Zebrafish Cranial and Trunk Neural Crest

**DOI:** 10.1371/journal.pone.0047394

**Published:** 2012-11-14

**Authors:** Erika Kague, Michael Gallagher, Sally Burke, Michael Parsons, Tamara Franz-Odendaal, Shannon Fisher

**Affiliations:** 1 Department of Cell and Developmental Biology, University of Pennsylvania, Philadelphia, Pennsylvania, United States of America; 2 Biology Department, Mount Saint Vincent University, Halifax, Nova Scotia, Canada; 3 McCusick–Nathans Institute of Genetic Medicine and Department of Surgery, The Johns Hopkins University, Baltimore, Maryland, United States of America; University of Sheffield, United Kingdom

## Abstract

The neural crest (NC) is a major contributor to the vertebrate craniofacial skeleton, detailed in model organisms through embryological and genetic approaches, most notably in chick and mouse. Despite many similarities between these rather distant species, there are also distinct differences in the contribution of the NC, particularly to the calvariae of the skull. Lack of information about other vertebrate groups precludes an understanding of the evolutionary significance of these differences. Study of zebrafish craniofacial development has contributed substantially to understanding of cartilage and bone formation in teleosts, but there is currently little information on NC contribution to the zebrafish skeleton. Here, we employ a two–transgene system based on Cre recombinase to genetically label NC in the zebrafish. We demonstrate NC contribution to cells in the cranial ganglia and peripheral nervous system known to be NC–derived, as well as to a subset of myocardial cells. The indelible labeling also enables us to determine NC contribution to late–forming bones, including the calvariae. We confirm suspected NC origin of cartilage and bones of the viscerocranium, including cartilages such as the hyosymplectic and its replacement bones (hymandibula and symplectic) and membranous bones such as the opercle. The cleithrum develops at the border of NC and mesoderm, and as an ancestral component of the pectoral girdle was predicted to be a hybrid bone composed of both NC and mesoderm tissues. However, we find no evidence of a NC contribution to the cleithrum. Similarly, in the vault of the skull, the parietal bones and the caudal portion of the frontal bones show no evidence of NC contribution. We also determine a NC origin for caudal fin lepidotrichia; the presumption is that these are derived from trunk NC, demonstrating that these cells have the ability to form bone during normal vertebrate development.

## Introduction

The evolution of vertebrates is concomitant with the evolution of the multi–potent neural crest (NC), which contributes to much of the vertebrate craniofacial skeleton. Therefore, an understanding of the evolution of the NC and in particular its contribution to the skeleton in different vertebrates lends insight into much broader questions of the origin of vertebrates. The current knowledge of the skeletogenic potential of the NC comes largely from studies of chicken and mouse development, with some key additional studies on other model organisms such as zebrafish and frog, and from these a broad consensus has emerged on several points. First, it is generally accepted that the cartilages of the pharyngeal arches are NC–derived. In the case of the mouse, long–term genetic lineage labeling has shown that the osteoblasts that replace these cartilages with bone, either directly (through endochondral ossification), or indirectly as adjacently forming membranous bones, are also derived from NC [1]. Second, it is clear that the bones in the vault of the skull are of mixed origin, with some being of NC origin and others deriving from head mesoderm [2], [3]. The exact boundaries are still somewhat uncertain, particularly in the avian embryo [3], [4]. Interestingly, data in the frog suggests that the entire vault of the skull contains NC-derived cells [5], unlike the situation in mouse or that supported by some of the data in chick. Finally, in neither mouse nor chick is there any evidence that trunk NC cells gives rise to cartilage or bone during normal development. Transplantation studies in chick have shown that trunk neural crest cells have skeletogenic potential, however this potential is not realized until these cells are put into the appropriate *in vitro* or *in vivo* environment [6], [7]. In zebrafish, Smith and colleagues demonstrated migration of trunk NC into the caudal fin mesenchyme [8]. The authors speculated these cells might contribute to the bony lepidotrichia, but lacked the lineage data to demonstrate that.

Aside from these areas of broad agreement, there are significant unresolved issues. Perhaps most importantly, thorough lineage studies with long–term labeling methods have only been performed in two species, the mouse and the chicken. It is likely misleading to extrapolate and assume the NC origin of specific aspects of the craniofacial skeleton in humans or other species. There also may be important contributions from the NC populations that are either transient or small, and require more careful investigation. For example, it has been suggested that small populations of NC cells are present in all sutures during formation of the mouse skull, and may even be required for proper suture patterning [9]–[11]. And while it seems clear that normally NC does not contribute to the skeleton caudal to the pectoral girdle in mouse or chicken, recent studies on the formation of the turtle carapace have challenged the assertion that trunk NC is not capable of forming bone and cartilage [12], [13].

While some studies on NC development in the zebrafish are in agreement with the broad consensus outlined above, there is currently no data from longer–term lineage studies that address the important issues of the origin of bones (as opposed to cartilages) in the craniofacial skeleton, or the skeletogenic potential of the trunk NC. Therefore, we have developed an approach to indelibly label the NC cells and their descendants, using a two–transgene system based on Cre recombinase. We can confirm results of previous lineage studies in the zebrafish, that demonstrated derivation of pharyngeal arch cartilages from NC [14]. In addition, we demonstrate NC origin for some later developing cartilage elements, and for many bones of the craniofacial skeleton. Interestingly, we find that only the most anterior portion of the vault of the skull is derived from NC, and that the posterior boundary falls within the frontal bone. Previous analyses of the pectoral girdle in other species had suggested that the cleithrum would be a bone of mixed origin, derived partly from mesoderm and partly from NC [15]; however, we find no evidence of NC contribution to the cleithrum in zebrafish. Finally, we show conclusively that the lepidotrichia in the caudal fin are derived from the NC, demonstrating that the trunk NC cells in zebrafish have realized their capacity to differentiate into osteoblasts (unlike in other model vertebrates). Most previous lineage studies have been carried out in amniotes; our results are critical in defining the characteristics of NC development that are particular to these groups, which characteristics are common to all vertebrates, which are unique to teleosts, and which may have been present in ancestral vertebrates. Furthermore, this study provides valuable insight into the study of neural crest evolution, providing support for the current thinking that fossil and extant lower vertebrates utilized trunk neural crest cells in the exoskeletal body coverings (dermal bone and dentine) unlike amniotes.

## Materials and Methods

### Ethics Statement

Fish were maintained according to standard protocols [16]. Studies were conducted in strict accordance with the Guide for the Care and Use of Laboratory Animals of the National Institutes of Health. The protocol #803318 was approved by the University of Pennsylvania Institutional Animal Care and Use Committee.

### Transgenic constructs

Two Cre reporter constructs were used in the work reported here. 1) The *ef1a*:*loxP-dsRed-loxP-egfp* (*egfp* reporter) construct was generated by cloning in a BamH1 fragment (*loxP-dsRed-polyA-loxP*) of *CMV:LoxP-dsRed-loxP-eGFP* (gift from Thomas Look [17]) upstream of the *egfp* gene in the T2KXIGΔIN vector (gift from Koichi Kawakami) at the available unique BamHI site. In the resulting transgenic fish the gene encoding fluorescent *dsRed* is expressed from the *ef1a* promoter/enhancer. Upon cre activation, expression is indelibly changed to *egfp*.

2) The *bactin:loxP-mcfp-loxP-hmgb1-mCherry* (*nucCh* reporter) construct was generated by cloning the promoter/enhancer region (5304 bp proximal to the ATG) from p5e-*bactin* plasmid (gift from Chi-Bin Chien) upstream of a Floxed cassette encoding a membrane tagged CFP (*mcfp*). Upstream of this cassette was cloned nuclear tagged mCherry (*nucCh*). Upon cre activation, expression is indelibly changed to *nucCh*.

The *-28.5Sox10:cre* was generated by cloning a previously described enhancer from upstream of the mouse *Sox10* gene [18] in front of the *cFos* minimal promoter and the *cre* coding sequence. Entry vector clones were constructed for the three components using the Tol2kit based on multi-site Gateway technology [19].

*-* ***210RUNX2:egfp*** **:** In a screen for cis–regulatory elements associate with *RUNX2*, we identified a conserved sequence from the last intron of the gene that acts to direct expression to early osteoblasts [20]. The enhancer was cloned upstream of the *cFos* minimal promoter and *egfp* in a Tol2 backbone to generate the *-210RUNX2:egfp* construct.

***-1.4col1a1:egfp*** **:** A 1.4 kb proximal promoter fragment of the zebrafish *col1a1* gene was cloned upstream of *egfp*, and the first intron of the gene cloned downstream, in a Tol2 vector backbone. Several independent transgenic lines demonstrated strong GFP expression in all cartilages, persisting into adult fish.

### Transgenic fish

Transgenic lines were generated via Tol2-mediated transgenesis, as previously described [21]–[23]. The Cre responder fish, carrying the *egfp* and *nucCh* reporters, are maintained as intercross stocks with multiple insertions. For the *Sox10:cre*, *-210RUNX2:egfp*, and *-1.4col1a1* fish, multiple independent lines were examined for each and showed similar patterns of expression (data not shown).

### Immunohistochemistry

Fish were fixed in 4% paraformaldehyde overnight at 4°C and stored in 0.01 M phosphate buffered saline, pH 7.4 (PBS) at 4°C until required. For whole–mount staining, larvae were washed in PBS plus 0.1% Tween-20 (PBST), transferred to methanol, and stored at −20°C at least overnight. After transfer back to PBST, larvae were digested briefly in Proteinase K and refixed in 4% PFA. After PBST washes and blocking in 10% goat serum, larvae were incubated with the 1° and 2° antibodies. For double staining, the 1° antibodies used were anti-GFP, 1∶500 (Invitrogen A11122) and anti-HuC, 1∶500 (Santa Cruz Biotechnology sc-56707) and the 2° antibodies were goat anti–rabbit IgG Alex Fluor488 conjugated and goat anti-mouse IgG AlexaFluor594 conjugated, both 1∶500.

For immunohistochemistry on sectioned tissue, frozen sections were cut at 15–20 µm and mounted on APTES (3-aminotriethoxysaline) coated slides. Tissue was incubated for one hour at room temperature in 10% bovine serum in PBS with 0.5% TritonX-100. The primary antibody used was anti-GFP (ABCAM AB6662) at a 1∶500 dilution. After incubation overnight at 4°C, tissues were mounted in a DAPI mountant (Vectashield sc24941).

### Photomicroscopy

For epifluorescence, live fish were anesthetized with Tricaine and observed and imaged on an Olympus MVX10 macroscope with mercury light source and filter sets for GFP and rhodamine. For examination of freshly dissected tissue, fish were euthanized by rapid immersion in ice water, immediately dissected, and tissue observed within 4 hours.

For confocal microscopy, live fish were anesthetized with Tricaine and mounted in glass bottom dishes with low melting point agarose in embryo medium. Samples were imaged on a Yokogawa spinning disc confocal; images were captured using Slidebook and processed with Image J.

## Results

### Cre expression in sox10 domain efficiently activates egfp reporter

Our goal was to express Cre recombinase broadly in the NC. We used a recently described enhancer associated with the mouse *Sox10* gene, which has been analyzed through transgenesis in mouse and zebrafish [18], [24]. The enhancer is located 28.5 kb upstream of *Sox10*, and in conjunction with a heterologous minimal promoter drives expression in NC and many known derivatives, including craniofacial cartilage, sympathetic ganglia, and enteric neurons. However, the *egfp* expression does not persist strongly in the embryo past 2 days post fertilization (dpf).

We constructed a transgene in which the same *Sox10* enhancer and minimal promoter are controlling *cre* expression (*-28.5Sox10:cre*). In preliminary experiments, we introduced the transgene into embryos also carrying a reporter transgene for Cre activity, in which the ubiquitous *ef1a* promoter is driving expression of *dsRed* flanked by LoxP sites, followed by *egfp* (*egfp* reporter). In the absence of an exogenous Cre transgene, dsRed is expressed strongly throughout the embryo, and no GFP+ cells are observed (data not shown). In many injected embryos, GFP was expressed mosaically, in cells apparently distributed as NC in the early embryo (data not shown). Injected fish were raised to adulthood and screened for germline transmission of both transgenes (*-28.5Sox10:cre* and the *egfp* reporter), as evidenced by embryos with GFP+ cells. Several independent lines were examined, and all yielded very similar patterns of expression.

**Figure 1:**
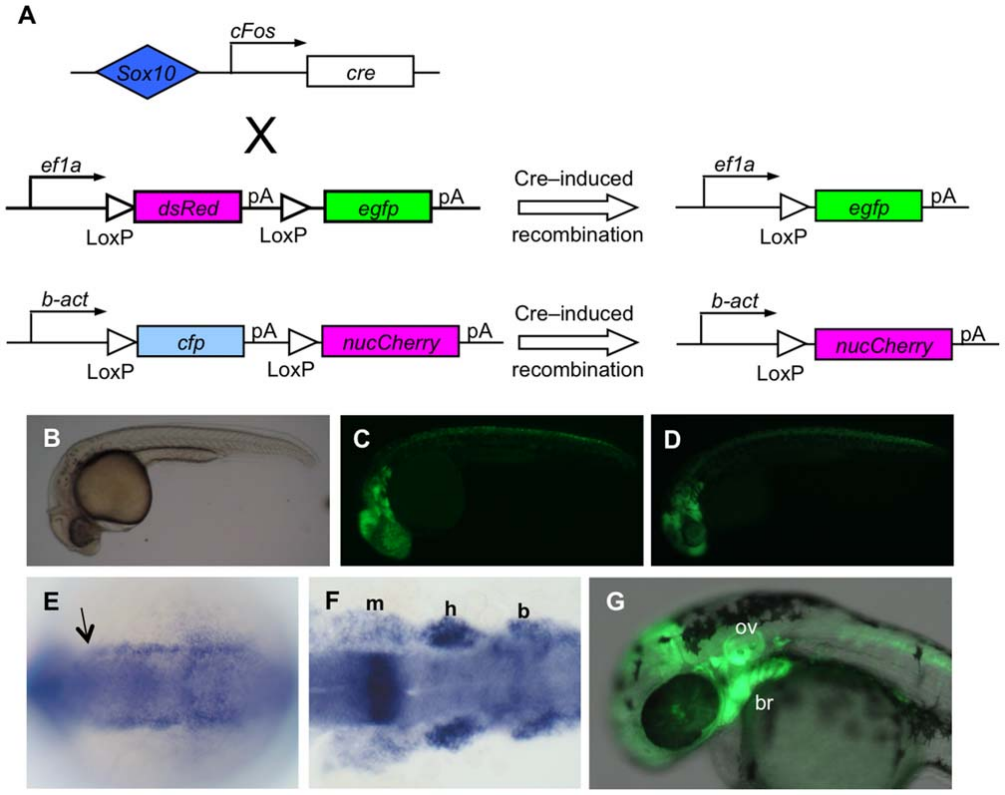
Cre recombinase permanently activates reporter gene expression in *sox10* expressing cells of the zebrafish embryo. A) Diagram of transgenes used to genetically mark neural crest descendants; Cre activity under control of the *Sox10* enhancer results in excision of the floxed first coding sequence in each reporter. In the first, *dsRed* is excised, leading to persistent expression of *egfp* under control of the ubiquitous *ef1a* promoter. In the second, *cyan fluorescent protein* (*cfp*) excision leads to persistent expression of nuclear *mCherry* (*nucCh*). B–D) At 24 hours post fertilization, *egfp* expression resulting from Cre activation (B, C) shows the same pattern as the expression under direct control of the *Sox10* enhancer (D). E, F) Expression of *cre* is shown by *in situ* hybridization of a *Sox10:egfp* transgenic embryo. E) Early expression of *cre* is seen to the anterior extent of NC (arrow) flanking the neural keel at 6 somites. F) Expression persists in the mandibular (m), hyoid (h), and branchial (b) clusters of NC at 14 somites. G) At 30 hours, doubly transgenic embryos show robust expression of *egfp* in cells known to be derived from neural crest, including in the branchial arches (br), and in the otic vesicle (ov). B–D, G are side views with anterior to the left; E and F are dorsal views with anterior to the left.

**Figure 2:**
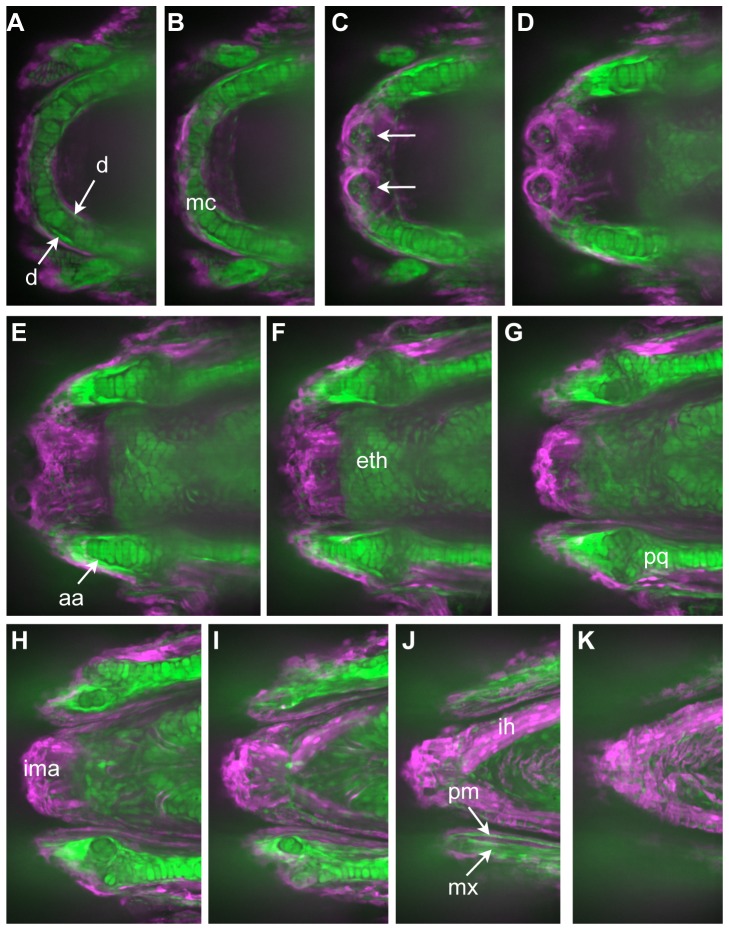
GFP expression persists and reveals pattern of neural crest derivatives in head. A–K represent successive Z-stack projections of five confocal sections each, moving from ventral to dorsal through the head of a 10dpf doubly transgenic embryo. Images have been colored so that green represents GFP+ cells (NC derivatives) and magenta dsRed+ cells (non-NC). Note that throughout the remaining figures, the label associated with NC (GFP or nucCherry) is always shown as green in the two–color overlays. Cartilages known to be NC-derived, including Meckel's cartilage (B), the ethmoid plate (F), and palatoquadrate (G) are labeled. Also GFP+ are cells in specific areas of ossification, including the dentary (A) and the anguloarticular (E) surrounding Meckel's cartilage, and the maxilla and premaxilla (J) of the upper jaw. Note also the GFP+ nerve plexus in the lip taste buds (arrows in C), representing their innervation by NC-derived cells of the facial ganglia. Non-NC-derivatives, such as the intermandibularis anterior (ima) and interhyoideus (ih) muscle masses, remain dsRed+. Abbreviations for skeletal structures are listed in Table 3.

**Figure 3:**
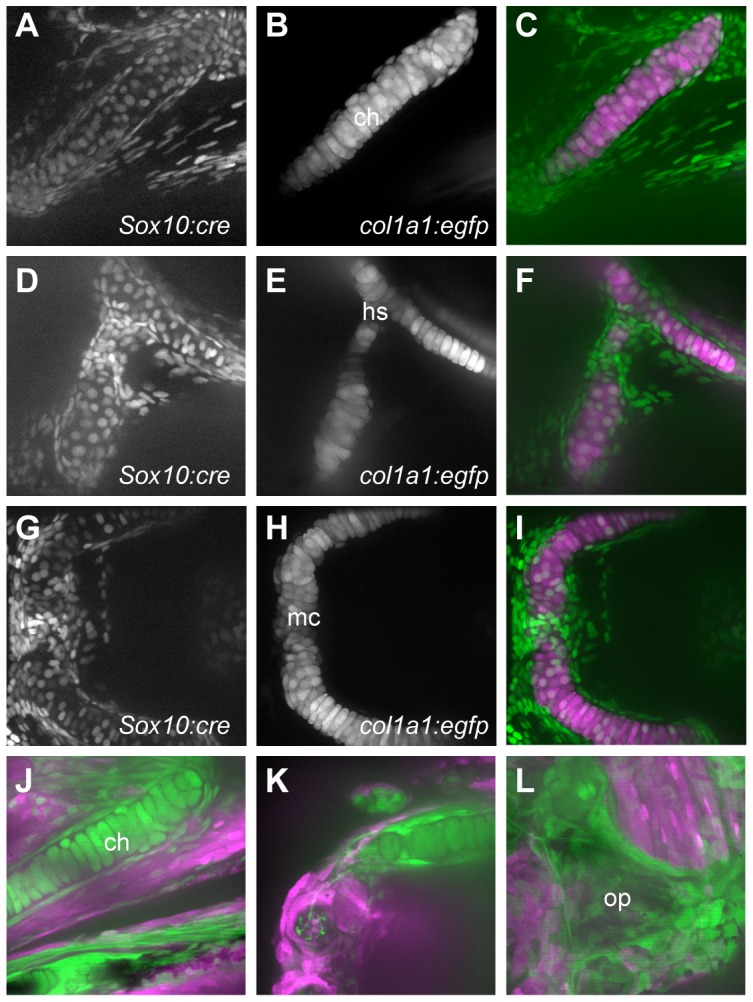
Chondrocytes and osteoblasts of the pharyngeal skeleton are NC-derived. A–I) Transgenics carrying a reporter that activates nuclear-Cherry expression following Cre activation (A, D, G) were crossed to *-1.4col1a1:egfp* transgenics, in which all cartilage cells are GFP+ (B, E, H). At 4 dpf, cells within the ceratohyal (A–C), hyosymplectic (D–F) and Meckel's (G–I) cartilages have nucCh+ nuclei, indicating they are NC-derived. The GFP− cells surrounding the cartilages, largely representing perichondral cells or osteoblast precursors, are also NC-derived. J–L) The reporter transgene switches from dsRed to GFP expression following Cre activation. At 10 dpf (J, K), cartilage cells of the ceratohyal (J) and Meckel's (K) cartilages are GFP+, as are the cells surrounding them, indicating that the bone replacing the cartilages is also NC-derived. Bones forming via membranous ossification, such as the opercle (L), are also NC-derived.

We examined *cre* expression directly by *in situ* hybridization, and find broad expression in cranial and trunk neural crest during somitogenesis (Fig. 1). At 24 hours post fertilization (hpf), GFP expression is seen in a distribution similar to the expression pattern regulated by the enhancer (Fig. 1), as previously described [18]. Cells are seen in the pharyngeal arches, and in the trunk migrating streams of NC cells are labeled by GFP expression. Although some of the founders are transmitting multiple copies of the *egfp* reporter transgene (data not shown), the conversion from dsRed to GFP expression seems nonetheless to be complete; we do not detect dsRed expression in GFP+ cells by confocal microscopy (e.g. Figs. 2, 3).

As an alternative reporter of Cre activity, we used a transgene with ß-actin promoter driving expression of *cyan fluorescent protein* (*cfp*) flanked by LoxP sites, followed by nuclear-localized *mCherry* (*nucCh* reporter). In fish doubly transgenic the *nucCh* reporter and the *Sox10:cre* transgenes, we observed the same pattern of nucCh+ cells as described above for the GFP+ cells (data not shown), indicating that both reporters accurately reflect Cre activity in the early embryo.

### Cells in peripheral nervous system are NC–derived

**Figure 4:**
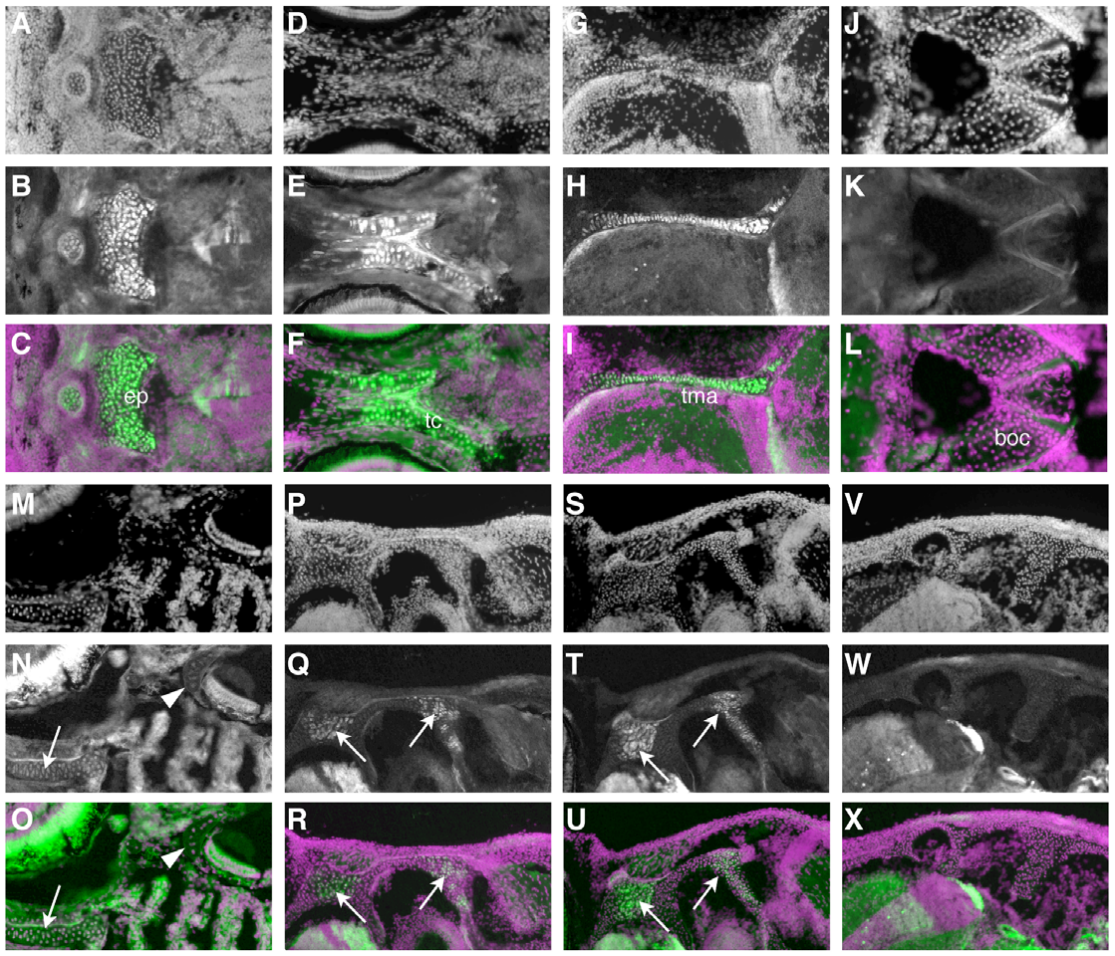
The chondrocranium is of mixed origin. A–R) Immunohistochemistry for GFP shows composition of cartilages with cellular resolution. In each set of three images, the first shows the DAPI counterstain (A, D, G, etc.), and the second (B, E, H, etc.) the GFP immunoreactivity. The third image in each group, the overlays, are pseudocolored with green representing GFP immunoreactivity and magenta the DAPI counterstain. The most anterior cartilages in the base of the skull, such as the ethmoid plate (A–C), trabeculae cranii (D–F), and taeniae marginalis anterior (G–I) are NC-derived. More posterior cartilages, like the basioccipital (J–L), contain no NC. M–O) A horizontal section at 14 dpf illustrates a more anterior NC-derived cartilage (arrow), the trabeculae cranii, and more posterior negative cartilage around the ear (arrowhead). P–X) Successive sections through a single fish at 44 dpf show that cartilage at intermediate locations, such as around the ear, is composed of a mix of NC (arrows) and non-NC cells in more ventral sections (P–R), and shows no NC-derived cells more dorsally (V–X).

The neurons of the dorsal root ganglia (DRGs) are known to be NC–derived in zebrafish, as in other organisms [25]. We find GFP+ cells in the DRGs, confirming that our genetic labeling includes these NC derivatives (Fig. S1A). Similarly, we find GFP+ cells in the hindgut (Fig. S1C), consistent with the known NC origin of the enteric neurons [26]. We performed double antibody staining for GFP and HuC, and in both cases we find the cells to be co–labeled, confirming their neuronal identity (Fig. S1B, D, E). There are additional cells in the DRGs, GFP+ but HuC−, which we presume are the NC-derived Schwann cells. Similarly, there are GFP+/HuC− cells in the intestine with the morphology of intestinal glial cells, also known to be NC–derived [27]. Within the cranial sensory ganglia, we find abundant GFP+/HuC+ neurons in the trigeminal, facial, and anterior and posterior lateral line ganglia (Fig. S1G–I, K). Although there are a few GFP+ cells in the vagal ganglia, these are not neurons, as evidenced by their failure to stain with anti-HuC (Fig. S1J, K). This is consistent with literature reporting NC contribution to neurons of the trigeminal, facial, and lateral line ganglia, but not to the vagal ganglia [14], [28], [29]. We also see a prominent GFP+ nerve plexus in the taste buds of the lip (Fig. 4C), presumably reflecting innervation by the facial nerve [30].

### Additional GFP+ progeny in double transgenic fish

In the hearts of our doubly transgenic fish, we find GFP+ cells within the myocardium, primarily in the region of the atrial-ventricular (AV) valve (data not shown). This is consistent with previous lineage data [31], [32], and also with the recently reported phenotype of a mutant in *leo1*, which has deficits in several NC lineages and a specific defect in cardiomyocyte differentiation in the AV valve region [33]. In the central nervous system, we also find that oligodendrocytes are GFP+ (data not shown), consistent with known expression of *sox10* in those cells. Melanoblasts are known to be NC–derived, and should also be GFP+ in the doubly transgenic fish. In early generations, in fish carrying multiple insertions of the *egfp* reporter transgene, we observed GFP+ melanoblasts (data not shown). However, in subsequent generations as the transgenes have been bred to single insertions, the melanoblasts are no longer labeled. This is consistent with the *ef1a* promoter displaying variable expression in specific differentiated cell types (M. P and S. F., unpub. obs.).

### NC contribution to the skeleton

**Figure 5:**
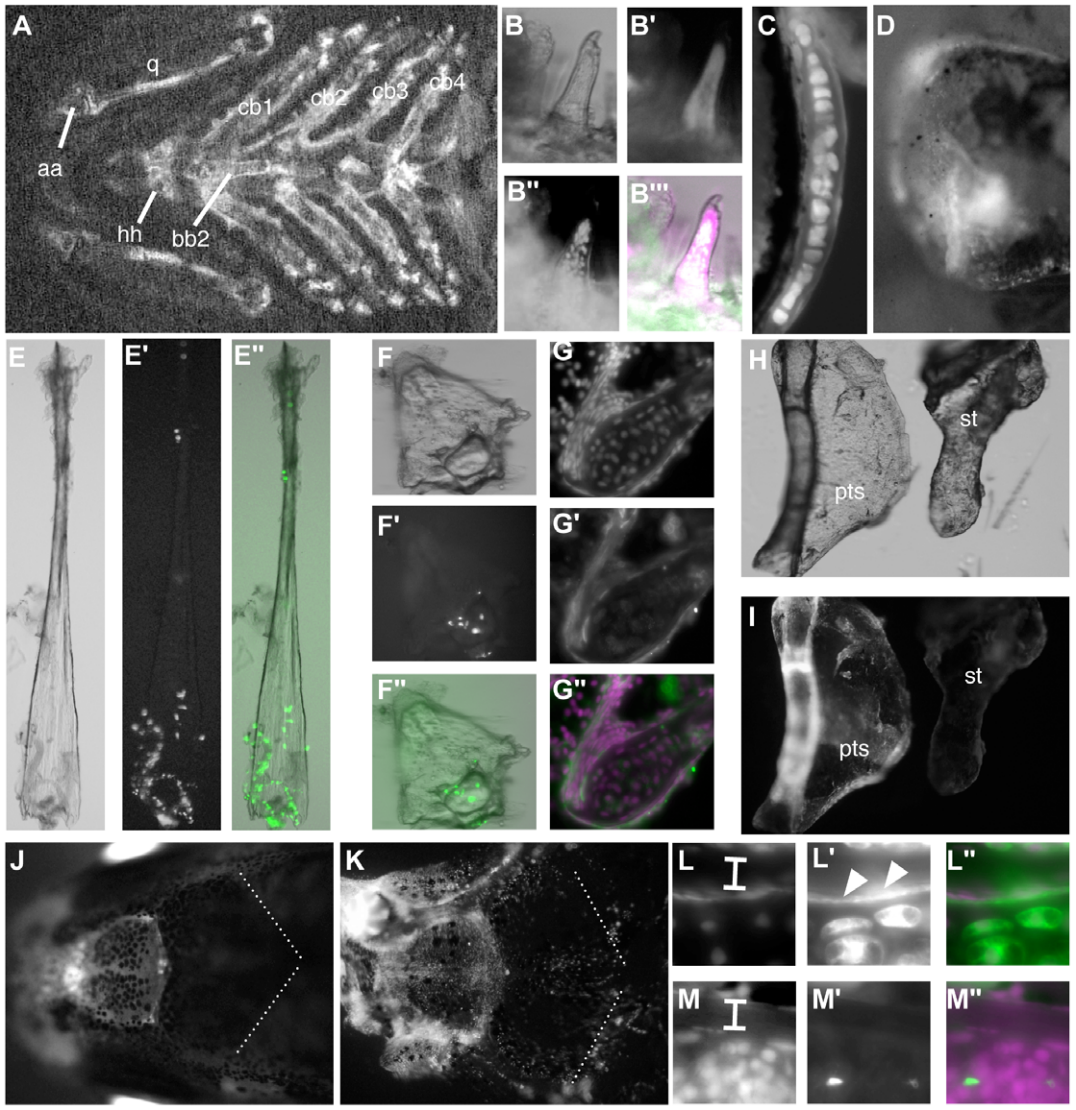
NC contribution to mineralized tissues of the adult skull. A) Bones derived by ossification of the pharyngeal arch cartilages are also NC-derived, seen in a horizontal section through a 44 dpf fish stained for GFP immunoreactivity. B) The odontoblasts of the pharyngeal teeth on the fifth ceratobranchial express the *RUNX2:egfp* transgene (B′) and are also NC-derived, seen by nucCh+ nuclei (B″). C, D) The scleral cartilages are NC-derived, shown by GFP immunohistochemistry (C), as are the ossicles derived by their ossification, seen as GFP+ in freshly dissected tissue (D). E) The parasphenoid shows no NC contribution, as seen in freshly dissected tissue. The scattered nucCh+ cells (E′, E″) are in associated soft tissue. The kinethmoid (F) also shows no NC contribution, although some of the associated soft tissue is NC-derived (nucCh+ in F′, F″). G-G″) Sections through the kinethmoid cartilage at 7 weeks show no GFP expression (G′) by immunohistochemistry (G is DAPI counterstain, G″ shows overlay). H, I) Dissections were used to determine the status of unresolved bones throughout the skull, e.g. the pterosphenoid is nucCh+ in freshly dissected tissue (I) and the supratemporal is not. J, K) The anterior portion of the frontal bones are NC-derived, seen as GFP+ cells in a live, 6-week-old fish (J), and also as nucCh+ cells in freshly dissected tissue (K). Dotted lines indicate the location of the coronal suture. The more posterior portion of the frontal bones, and the other flat bones of the skull, show no evidence of NC contribution. L–M″) Sections through the anterior frontal bone of a 7-week fish (L-L″) show GFP+ osteoblasts by immunohistochemistry (arrowheads in L′) aligned under the acellular bone matrix (bracket in L), as well as GFP+ cartilage cells in the underlying epiphyseal bar. A similar section through the posterior frontal bone (M-M″) shows no GFP expression in the osteoblasts (M′). In each set of panels, the first is the DAPI counterstain, the second the GFP immunohistochemistry, and the third the overlay.

**Figure 6:**
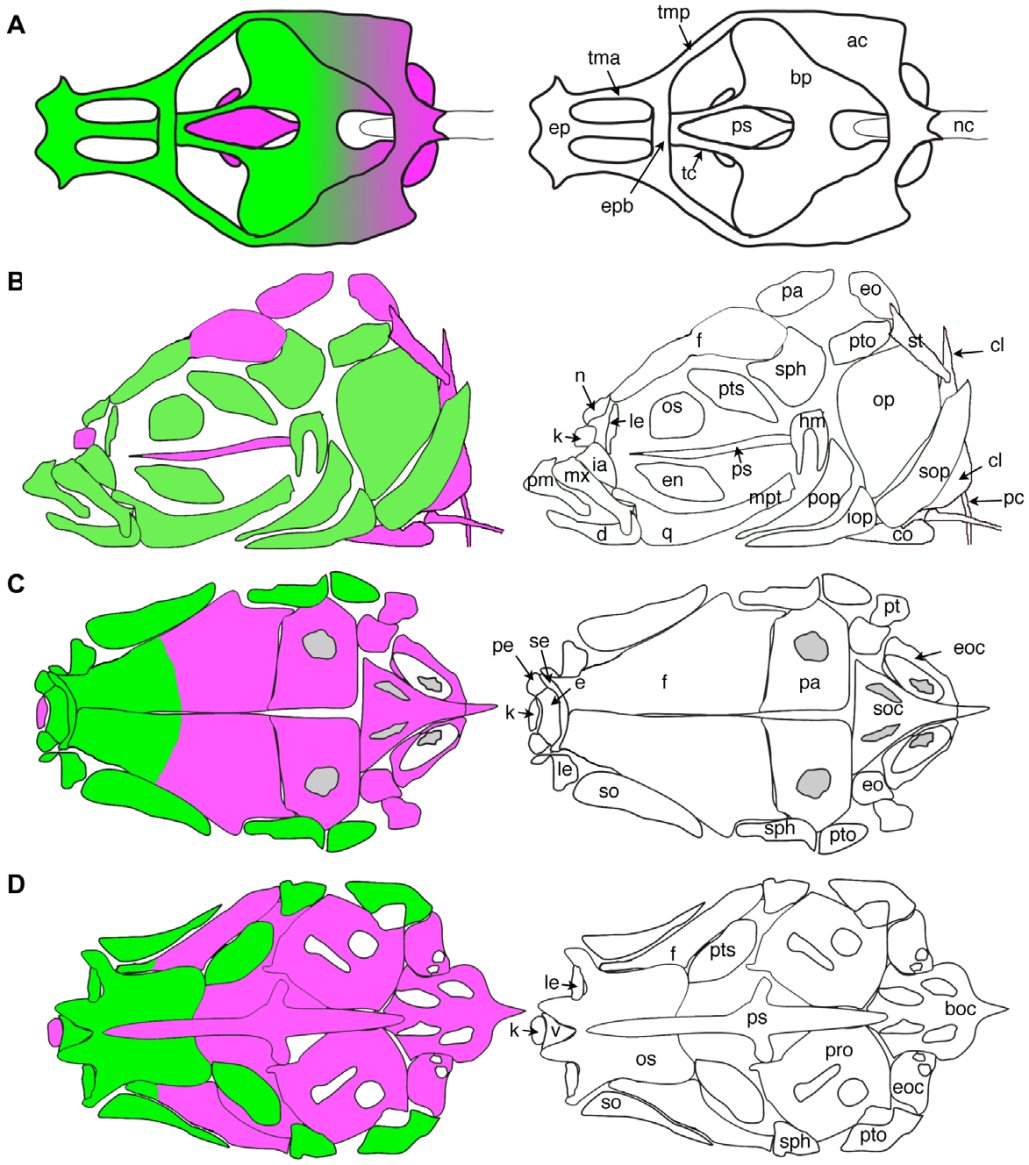
Limits of NC contribution to the zebrafish craniofacial skeleton. Diagrams depict the cartilage elements and bones that are NC-derived (green), and those that show no evidence of NC contribution, and are presumably derived from mesoderm (magenta). The diagram in A shows a dorsal view of the chondrocranium from an approximately 12 dpf larva. B is a side view of the bones of an adult skull, with some elements of the pectoral girdle also shown. C shows a dorsal view of the dorsal aspect of the adult skull. In D, the view is of the base of the neurocranium, with the pharyngeal skeleton removed. Skeletal elements are labeled according to the abbreviations in Table 3. Note that in all diagrams, some elements are omitted for the sake of clarity; drawings were modified from Cubbage and Mabee (1996) and [62].

We have classified the skeletal elements of the craniofacial skeleton and pectoral girdle with respect to their embryological origin (NC-derived or not) through a combination of observations of live transgenics via epifluorescence or confocal microscopy; immunohistochemistry for GFP on sectioned tissue; and freshly dissected tissue imaged via epifluorescence. Below we discuss specific examples, with data shown in Figures 2, 3, 4, and 5; our overall results are summarized in Figure 6, and in Tables 1 and 2.

**Table 1:** Neural crest contribution to cartilage elements in the craniofacial skeleton.

Region	Cartilage	Neural crest
**Anterior arches**	Meckel's cartilage	Y
	palatoquadrate	Y
	basihyal	Y
	ceratohyal	Y
	hyosymplectic	Y
	interhyal	Y
**Branchial arches**	Ceratobranchials (1–5)	Y
	Hypobranchials (1–4)	Y
	Basibranchials (1–4)	Y
	Epibranchials (1–5)	Y
	Pharyngobranchials (1, 2+3)	Y
**Neurocranium**	Ethmoid plate	Y
	Trabeculae cranii	Y/N
	Parachordal/basal plate	Y/N
	Anterior basicranial commissure	Y
	Posterior basicranial commissure	Y
	Occipital arch	N
	Sclerotic cartilages	Y
	Kinethmoid	N
**Pectoral girdle**	Coraco-scapular	N
	Mesocoracoid	N
	Proximal radials (four)	N
	Distal radials (four)	N
	Propterygium	N
	Basipterygia	N

For each cartilage element, we indicate whether we observed contribution by neural crest descendants (Y = yes; N = no); Y/N indicates cartilages that appear to be of mixed origin.

**Table 2:** Neural crest contribution to craniofacial bones.

Region	Bone	Type	Neural crest
**Olfactory**	ethmoid	c	Y
	kinethmoid	c	N
	lateral ethmoid	c	Y
	nasal	d	Y
	preethmoid	c	Y
	supraethmoid	d	Y
	vomer	d	Y
**Orbital**	frontal	d	Y/N
	infraorbitals	d	Y
	orbitosphenoid	c	Y
	parasphenoid	d	N
	pterosphenoid	c	Y
	sclerotic, anterior	c	Y
	sclerotic, posterior	c	Y
	supraorbital	d	Y
**Otic**	epioccipital	c	N
	parietal	d	N
	posttemporal	d	N
	prootic	c	Y
	pterotic	c	Y
	sphenotic	c	Y
	supratemporal	d	N
**Occipital**	basioccipital	c	N
	exoccipital	c	N
	notochord	pc	N
	supraoccipital	c	N
**Mandibular arch**	anguloarticular	d	Y
	coronomeckelian	d	Y
	dentary	d	Y
	maxilla	d	Y
	premaxilla	d	Y
	retroarticular	c	Y
**Palatoquadrate arch**	ectopterygoid	d	Y
	entopterygoid	d	Y
	metapterygoid	c	Y
	palatine	c	Y
	quadrate	c	Y
**Hyoid arch**	basihyal	c	Y
	branchiostegal rays (1–3)	d	Y
	ceratohyal	c	Y
	epihyal	c	Y
	hyomandibula	c	Y
	hypohyal, dorsal	c	Y
	hypohyal, ventral	c	Y
	symplectic	c	Y
	urohyal	d	Y
**Branchial arches**	basibranchial (1–3)	c	Y
	ceratobranchial (1–5)	c	Y
	ceratobranchial 5 teeth		Y/N
	epibranchial (1–4)	c	Y
	hypobranchial (1–3)	c	Y
	pharyngobranchial (1, 2+3)	c	Y
**Opercular**	interopercle	d	Y
	opercle	d	Y
	preopercle	d	Y
	subopercle	d	Y
**Pectoral girdle**	cleithrum	d	Y
	coracoid	c	N
	mesocoracoid	c	N
	postcleithrum	d	N
	radials	c	N
	scapula	c	N
	supracleithrum	d	N

For each bone, we indicate mode of ossification (c = cartilage replacement; d = dermal; pc = parachordal), and whether we observed contribution by neural crest descendants (Y = yes; N = no); Y/N indicates bones that appear to be of mixed origin.

**Table 3:** List of abbreviations for skeletal elements.

aa	anguloarticular	mr	median ray
ac	auditory capsule	mx	maxilla
bb	basibranchials	n	nasal
bh	basihyal	nc	notochord
boc	basioccipital	op	opercle
bp	basal plate	os	orbitosphenoid
bsr	branchiostegals	p	palatine
cb	ceratobranchials	pa	parietal
ch	ceratohyal	pb	pharyngobranchials
cl	cleithrum	pc	postcleithrum
cm	coronomeckelian	pe	preethmoid
co	corocoid	pm	premaxilla
d	dentary	pop	preopercle
e	ethmoid	pro	prootic
eb	epibranchials	ps	parasphenoid
ect	ectopterygoid	pt	posttemporal
eh	epihyal	pto	pterotic
en	entopterygoid	pts	pterosphenoid
eo	epioccipital	q	quadrate
eoc	exoccipital	ra	retroarticular
ep	ethmoid plate	sa	sclerotic, anterior
epb	epiphyseal bar	se	supraethmoid
f	frontal	so	supraorbital
hb	hypobranchials	soc	supraoccipital
hhd	hypohyal, dorsal	sop	subopercle
hhv	hypohyal, ventral	sp	sclerotic, posterior
hm	hyomandibula	sph	sphenotic
ic	intercalar	st	supratemporal
inf	infraorbitals	sy	symplectic
iop	interopercle	tc	trabeculae cranii
k	kinethmoid	tma	taenia marginalis anterior
le	lateral ethmoid	tmp	taenia marginalis posterior
mpt	metapterygoid	v	vomer

### Cartilage and bones of the viscerocranium are NC–derived

Known derivatives of NC are GFP+ in the doubly transgenic embryos, including cartilages of the viscerocranium, derived from the pharyngeal arches (Fig. 2, 3). The cartilages of the pharyngeal arches are the earliest craniofacial skeletal elements to form, visible morphologically beginning at 2 dpf, and they have been previously shown to be entirely derived from NC of the mesencephalon and hindbrain rhombomeres [14]. We also find that these cartilages are entirely NC-derived (Figs. 2, 3). Notably, we do not see evidence of mosaic activation of the reporter transgene in cartilage; because of its distinctive morphology and large cell size, it is quite easy to see unlabeled cells. In the few fish where GFP expression was mosaic, the expression of the *dsRed* reporter transgene was also mosaic (data not shown), suggesting that the Cre activity is quite robust from the *-28.5Sox10:cre* transgene.

Many of the cartilages of the viscerocranium are converted to bone through perichondral ossification, over a period of many weeks. The mineralized bone begins to accumulate at 5–6 dpf, visible by staining with calcium chelators, in collars around the cartilage elements. We find that the perichondral cells surrounding cartilages that ossify in this manner, such as the ceratohyal, are NC–derived (Fig. 3). Some other bones, notably the dentary and anguloarticular of the lower jaw, and the maxilla and premaxilla of the upper jaw, form via intramembranous ossification. The cells adjacent to these cartilages in the early larva, prior to ossification but in the locations where ossification will later take place, are also NC–derived (Fig. 3), as are the specific ossifications at later larval stages, when they can be distinguished (Fig. 4).

The opercle develops by intramembranous ossification, in close apposition to the hyosymplectic cartilage. Although the opercle was presumed to also be derived from NC of the second pharyngeal arch, it had not been directly demonstrated by lineage. We find that the opercle is NC derived (Fig. 3L), as are the branchiostegal rays (data not shown); these are also membranous bones likely to be derived from cells of the branchial arches based on their position.

### The neurocranium is of mixed origin

Some elements that contribute to the base of the skull begin to form quite early, within the first week, including the parachordal cartilages and parasphenoid bone. The parachordals form by condensation around the anterior tip of the notochord, and do not show evidence of NC contribution (data not shown). The more anterior ethmoid plate and attached trabeculae cranii are NC-derived (Figs. 2, 4A–F), consistent with previous lineage studies [34]. Eventually, the trabeculae join the parachordals posteriorly, and are contiguous with them as they expand to form the basal plates. At these later stages, we find that the cartilage in the more posterior regions of the ventral neurocranium is a mixture of NC and non-NC cells (Fig. 4P–X). Surprisingly, the more anterior parasphenoid is not NC-derived (Fig. 5E-E″). The most anterior bone that is not NC-derived is the kinethmoid (Fig. 5F-F″), a small midline bone at the anterior tip of the upper jaw, that forms as a sesamoid bone within the intermaxillary ligament [35]; the cartilage within which it ossifies is also not labeled (Fig. 5G-G″).

In the zebrafish, the flat bones of the skull (frontal, parietal, exoccipital bones) form several weeks after the facial bones, relatively late compared to the same process in the mouse. In live transgenic fish at 6 weeks, shortly after the flat bones have met at the coronal and sagittal sutures, GFP expression can be seen in the vault of the skull in the anterior portion of the frontal bones, with its posterior border at the position of the underlying epiphyseal bar cartilage (Fig. 5J). The posterior portion of the frontal bones, as well as the parietal and occipital bones, are GFP−. We verified this finding through dissection of fresh, unfixed tissue from a fish carrying the *nucCh* reporter transgene, and again observed that nucCh+ cells were confined to the anterior portion of the frontal bone (Fig. 5K). Immunohistochemistry for GFP on sectioned material confirms that the osteoblasts of the anterior frontal bone are labeled, while those in more posterior regions are not (Fig. 5L–M″)

### The cleithrum does not contain neural crest–derived cells

**Figure 7:**
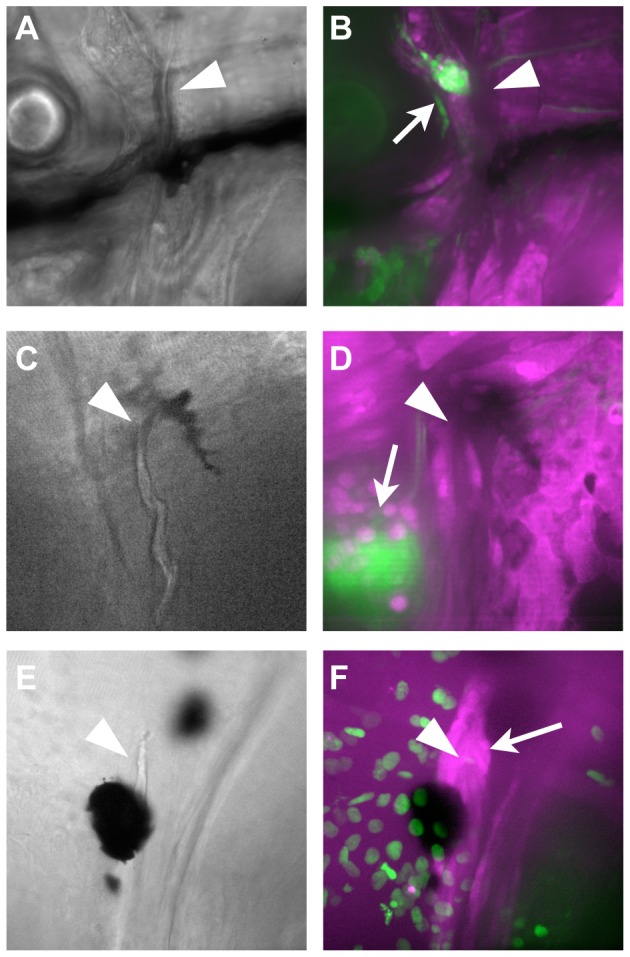
NC does not contribute to the cleithrum. A–D) At 10 dpf (A, B) and 16 dpf (C, D), Z-stack projections of confocal sections through the area surrounding the dorsal end of the cleithrum (arrowheads) reveal no GFP+ cells. The NC-derived glia of the lateral line ganglia are clearly visible in the same fields of view (arrows). E, F) To localize the osteoblasts, fish carrying the *nucCh* reporter were crossed with *RUNX2:egfp* transgenics, in which the osteoblasts are GFP+. The dorsal tip of the cleithrum (arrowhead in E, F) is surrounded by osteoblasts (arrow in F), which do not have nucCh+ nuclei, indicating they are not NC-derived.

The pectoral girdle represents a transition area in vertebrates between the portion of the skeleton derived from NC and that from mesoderm. In particular, the cleithrum had been predicted previously to be of mixed origin (i.e. partially NC-derived), much as the clavicle is in mammals [15], based on the embryological origins of the associated muscle attachments. In the juvenile fish at six weeks, we find GFP+ cells associated with the most dorsal tip of the cleithrum, visible when the bone is dissected (data not shown). However, they are not in the bone, but in the associated soft tissue. We examined the cleithrum more closely during its formation, by confocal microscopy. At stages from 16 to 21dpf, we can observe no NC cells associated with the dorsal tip of this bone (Fig. 7A–D). The osteoblasts associated with the bone at this stage are difficult to identify by morphology and position alone. Therefore, we also examined the cleithrum in a *RUNX2:egfp* transgenic line, in which early osteoblasts are GFP+. At 21 dpf, the osteoblasts are clustered around the tip of the bone (Fig. 7E, F); they do not have nucCh+ nuclei, which mark the NC derivatives in the same fish.

### Neural crest contributes to the post-cranial skeleton

**Figure 8:**
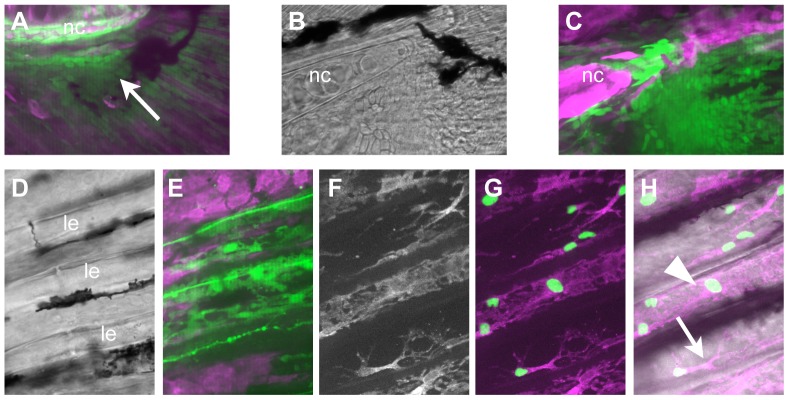
The scleroblasts of the caudal fin are NC derived. A) At 8 dpf, NC-derived cells (GFP+; arrow) can be seen clustered around the tip of the notochord (nc). B, C) By 16 dpf, there are more GFP+ cells; some are located more distally in the fin, although many are still close to the notochord. D–H) At 21 dpf, the caudal fin contains well-formed lepidotrichia (le in D), which are associated with GFP+ cells (E). F–H) To confirm the identity of the cells as osteoblasts, fish carrying the *nucCh* reporter were crossed with *RUNX2:egfp* transgenics, in which the osteoblasts are GFP+. The osteoblasts have nucCh+ nuclei, indicating they are NC-derived (G), and they are located both within (arrowhead) and immediately outside (arrow) the lepidotrichia (H).

The *Sox10* enhancer regulating expression of *cre* is also active in the trunk neural crest, allowing us to examine their contribution to tissues at later stages. In the caudal fin fold, we observed GFP+ cells clustered around the tip of the notochord as early as 2 dpf (data not shown); by 8 dpf, the accumulation is more striking, and comprises a group of ∼200 cells (Fig. 8A); the nearby hypural cartilages do not contain labeled cells. By 16 dpf, the forming lepidotrichia, or bony fin rays, are visible, and by 21 dpf a pattern essentially similar to the adult fin is formed. At these stages, the NC-derived cells associate with the lepdotrichia, and based on position and morphology appear to be osteoblasts (Fig. 8B–E); we have observed similarly positioned labeled cells in the dorsal fin, although the pectoral fin lepidotrichia are not labeled (data not shown). To confirm their identity, we again examined the *RUNX2:egfp* transgenic fish. At 21 dpf, we observed GFP+ cells both within the hollow lepidotrichia and closely associated with the outside surface, where osteoblasts are known to be located. These cells also have nucCh+ nuclei, indicating their NC origin (Fig. 8F–H).

## Discussion

Several reports have demonstrated the usefulness of Cre recombinase in zebrafish for activating transgenes in specific cell types, both as a means of misexpression, for example of oncogenes [17], [36], [37], and of lineage tracing [38]–[41]. In this study, we have primarily addressed the question of the embryological origins of skeletal elements in the zebrafish. Because of the prolonged phase of pre–metamorphic development and late formation of the adult body form, diverse elements of the zebrafish skeleton are formed over a period of approximately six weeks, far longer than the period encompassing similar events in the mouse or chicken. While the lineage of some elements, such as those in the viscerocranium, can be determined by non–genetic methods, others require an indelible method of lineage tracing. Therefore, we developed a two–transgene system based on Cre recombinase, and used it here to examine the derivation of skeletal elements into the adult, as well as other cell types derived from NC.

In non–skeletal tissues, our results are largely consistent both with what is known in zebrafish, and data from other model organisms. We find many cells of the peripheral nervous system are NC–derived, including Schwann cells and neurons of the DRGs, enteric neurons, and neurons of some cranial sensory ganglia. In the mouse and chick, cardiac NC is important for proper patterning of the aortic arches, and directly contributes to the septum dividing the right and left outflow tract [42]. Since zebrafish has a two–chambered heart, it was unclear what role NC would play in heart development. Indeed, previous reports of NC contribution to the zebrafish heart suggested that it differentiated into myocardium [31], [32]. Consistent with these findings, we find NC contribution to cells within the myocardium, primarily in the AV valve region. Our finding is also consistent with the reported phenotype of a mutant in *leo1*, encoding a member of a complex of proteins active in chromatin remodeling [33], and support a mechanism for the cardiac defects arising directly from NC deficits.

In other vertebrates for which there is lineage evidence available, the cartilage elements of the pharyngeal arches are NC-derived. In the zebrafish, these cartilages develop within the first several days post fertilization [43], making them amenable to classic lineage tracing via dye injection [14]. These earlier lineage studies, performed at the level of single cells, demonstrated that all cartilages comprising the pharyngeal arch skeleton are NC-derived. Interestingly, they demonstrated that most premigratory NC cells were already lineage restricted, and gave rise to clones of a single cell type. They did not analyze these cells with markers for specific cell types, or at a late enough stage to examine osteoblast lineage; osteoblasts would presumably have fallen into their “unidentified” cell pool. Together with our data that perichondral cells and osteoblasts are NC-derived, these earlier lineage studies might suggest that while chondrocytes and osteoblasts share NC lineage, they are derived from separate precursor cells, which are specified prior to NC migration.

We confirm the general vertebrate pattern in formation of the chondrocranium in zebrafish, that more anterior cartilage elements are NC-derived, while more posterior elements have a mixed origin, presumably with a mesodermal contribution. A particularly thorough study of this issue has been carried out in the mouse [44]; these authors identify a few cartilages with a mixed origin. However, in contrast to our finding of substantial mixing of NC and non–NC cells, they report distinct boundaries between the NC and mesoderm–derived portions of these elements. We speculate that this is because the separate centers of chondrification in the zebrafish fuse relatively early, and grow substantially after fusion.

A number of zebrafish mutants with craniofacial abnormalities have been identified in large–scale genetic screens [45]–[47]; based on our lineage results, we would predict that a mutant either lacking neural crest, or with a failure of neural crest development into cartilage, would show defects in only the ventral and anterior portions of the chondrocranium. While no such single mutant has been described, in fish deficient for both *foxd3* and *tfap2a* the neural crest apparently fails to differentiate into cartilage [48]. These larvae retain the most posterior portion of the neurocranium, and lack all other craniofacial cartilages, consistent with our results.

We find that the bones in the base of the skull are of mixed origin; most surprisingly, we find no evidence of NC contribution to the parasphenoid, which in the adult extends to the rostral border of the eye. The literature is somewhat unclear about the homologies between bones in the base of the neurocranium in zebrafish and in other vertebrates, although the midline bone in the equivalent position in the mouse (referred to variously as the “presphenoid” and the “parasphenoid” by different authors) is neural crest derived [44]. The parasphenoid in chicken has been described as mixed in origin, with NC contribution anteriorly, while the posterior is derived from somitic mesoderm [3], [47]. In *Xenopus laevis*, the parasphenoid is at least partially NC-derived [49], although the nature of the labeling procedure makes it impossible to rule out a mesodermal contribution.

We also find that another very anterior bone, the kinethmoid, is not of NC origin. The ontogeny of the kinethmoid is atypical; it forms, relatively late, as a sesamoid cartilage element, embedded entirely within the intermaxillary ligament, similar to the patella in humans [35]. It is also unique to cypriniforms and lacking in other fishes [50]. We speculate that because of its late and atypical ontogeny, it is derived from a pool of precursor cells distinct from those that give rise to the earlier patterned cartilages of the pharyngeal arches and neurocranium.

The flat bones of the skull develop relatively late in the zebrafish; the frontal and parietal bones first become visible as small areas of ossification around 3–4 weeks post-fertilization, and grow to meet at the sutures around 6 weeks in wild–type fish [43], [51], and our own observations). Given this lag in formation, only a genetic method of lineage tracing would allow determination of the embryological origin of the bones. Consistent with results from mouse [2], [15], [52], we find that the more posterior bones, the exoccipital and the paired parietal bones, do not have a NC contribution. Additionally, we find that only the anterior portions of the paired frontal bones are derived from NC, and there is a clear anterior-posterior boundary where the epiphyseal bar cartilage passes underneath the frontal bones. In *Xenopus laevis*, the best evidence is that NC contributes to the entire anterior-posterior extent of the frontoparietal bones [5], although it is possible that there is also a contribution from mesoderm. The derivation of the frontal bones in the chicken has been disputed in the literature [53], although recent retroviral-based lineage studies suggest that, as in the zebrafish, the frontal bone is of mixed origin, with only the anterior portion derived from NC [4]. Given these species differences, it is difficult to reconstruct what might have been the ancestral vertebrate derivation of the skull vault. Even disregarding the conflicting data in chicken, the remaining evidence suggests two extremes, with amphibians having an entirely NC-derived skull vault, while zebrafish have only a small anterior NC contribution.

The pectoral girdle is a complex area of the skeleton, with contributions from mesoderm and neural crest described in different vertebrates. Bones form by a mixture of membranous and endochondral ossification, and what are thought to be analogous bones in different species sometimes ossify via different modes [20], [46]. Finally, there is at least one bone, the clavicle in the mouse, that both forms by a combination of membranous and endochondral ossification [54], [55], and is derived partially from NC and partially from mesoderm [15]. It is difficult in some cases to assign direct analogies between bones in different species, and different elements of the ancestral pectoral girdle complex have been preserved in different lineages through evolution. It has been argued that the pattern of muscles in the region, and their associated attachments to skeletal elements, are more highly conserved [15], although that assertion has been disputed [56], [57]. Furthermore, there is evidence in the mouse of a correlation between the embryological origin of the attachments themselves, whether from NC or mesoderm, and the origin of the associated bones [15]. Based on these observations, it was predicted that the cleithrum in bony fish would be a bone of mixed origin, similar to the clavicle. However, we examined the cleithrum through the first three weeks of development, at the resolution of single cells through confocal microscopy, and failed to see any contribution of NC cells; indeed, we find that the entire chain of bones connecting the pectoral girdle to the skull is non–NC in origin. We cannot completely rule out a later contribution, but at the gross level in dissected tissue, there did not appear to be any contribution at six weeks (data not shown).

The membranous bones of the fins, the lepidotrichia, develop relatively late in the zebrafish. Early fin folds have collagenous actinotrichia, arranged radially, which provide structure to the fin fold and likely serve in some way as a scaffold for later formation of the bones. In the caudal fin, the first appearance of the bones is at ∼4.9 mm standard length, or approximately 10 dpf in normal fish [58]. The bones are made by scleroblasts (the functional equivalent of osteoblasts in the fin), located both outside (in less mature bone) and within the bone matrix of the hollow lepidotrichia [59]. The derivation of these cells during fin ontogeny has not been described previously; we show here that they are derived from NC. We see NC-derived cells located near the tip of the notochord during late larval and early juvenile development, but few of these appear to enter the fin fold. At 16 dpf, we see substantial evidence of these cells invading the fin, coincident with extensive bone formation. Finally, at 21 dpf, when essentially the adult pattern of lepidotrichia is established, the NC progeny are associated with the bones and expressing a marker of osteoblasts. Based on experimental evidence in chicken and mouse, it has long been a tenet that post–cranial NC does not contribute to the skeleton during normal development, although several studies have suggested that trunk NC cells have skeletogenic potential which is only realized when they are placed in a permissive environment [7]. Interestingly, recent studies in a non–traditional model organism, the turtle [12], [13], [60] suggest that the plastron bones in the carapace are derived from a late–emerging population of trunk neural crest. Together with our own results, this lends support to a model where the ability of the trunk NC to form skeletogenic tissues was the ancestral condition; this ability was lost in disparate lineages concomitant with the loss of exoskeletal body armor and other intramembranous bones of the post–cranial skeleton.

In several instances, our data point to a non-NC origin for bones that appear to be NC-derived in other vertebrates. For example, we find that the parasphenoid in the base of the neurocranium is not NC-derived, although the homologous bones in mouse, chicken, and amphibians appear to be, at least partially. We also find that the frontal bones in zebrafish are of mixed origin, although they are entirely NC-derived in the mouse and possibly also in amphibians, while the situation is less clear in the chicken. And although the cleithrum is not directly homologous to any bone in mammals, it was predicted that it would be of mixed origin; however, we find no NC contribution. While overall we largely find conservation in the composition of craniofacial skeletal elements between fish and amniotes, our results also suggest that in some regions the specific origin of bones in the skull is fluid, where there are two populations of cells with the potential to form bone or cartilage, and the composition of homologous bones in different species can depend on fairly subtle variations in cell number or the exact location and strength of inducing signals. A similar idea has been proposed based on heterotopic avian NC grafts [61], in which transplanted NC cells in sufficient numbers were capable of participating in the formation of morphologically normal cartilages which would normally be mesodermal in origin. It is interesting to speculate how such a situation could have evolved, since the embryological origin and development of the neural crest and the mesoderm is so dramatically different.

## Supporting Information

Figure S1 **Cells of the peripheral nervous system are NC-derived.** A, B) Combined GFP/HuC immunostaining reveals that neurons of the DRG are GFP+ (A) and HuC+ (B); there are also GFP+/HuC− cells visible in some ganglia (arrows), presumably Schwann cells. C–E) Enteric neurons are GFP+ (C) and HuC+ (E); in all panels with merged images (D, G–K), GFP is shown in green and HuC in magenta. There are also some GFP+/HuC− cells, which may represent NC-derived enteric glial cells (arrowheads). F) Antibody staining for HuC reveals neurons of the cranial sensory ganglia in a 4 dpf larva. In the trigeminal (G), facial (H), anterior lateral line (I), acoustic (I) and posterior lateral line (K) ganglia, there are numerous doubly positive neurons, indicating substantial NC contribution. In contrast, in the vagal ganglia, there are only a few GFP+ cells, which are not HuC+ (J, K). All images in D and G–K are single confocal slices. Abbreviations: a (acoustic ganglion); all (anterior lateral line ganglion); f (facial ganglion); pll (posterior lateral line ganglion); tg (trigeminal ganglion); v (vagal ganglia).
(TIF)
