# Supplementary figures and images for: Skeletogenic Fate of Zebrafish Cranial and Trunk Neural Crest
Source: PLoS One. 2012 Nov 14;7(11):e47394. doi: 10.1371/journal.pone.0047394 (PMC3498280; doi:10.1371/journal.pone.0047394)

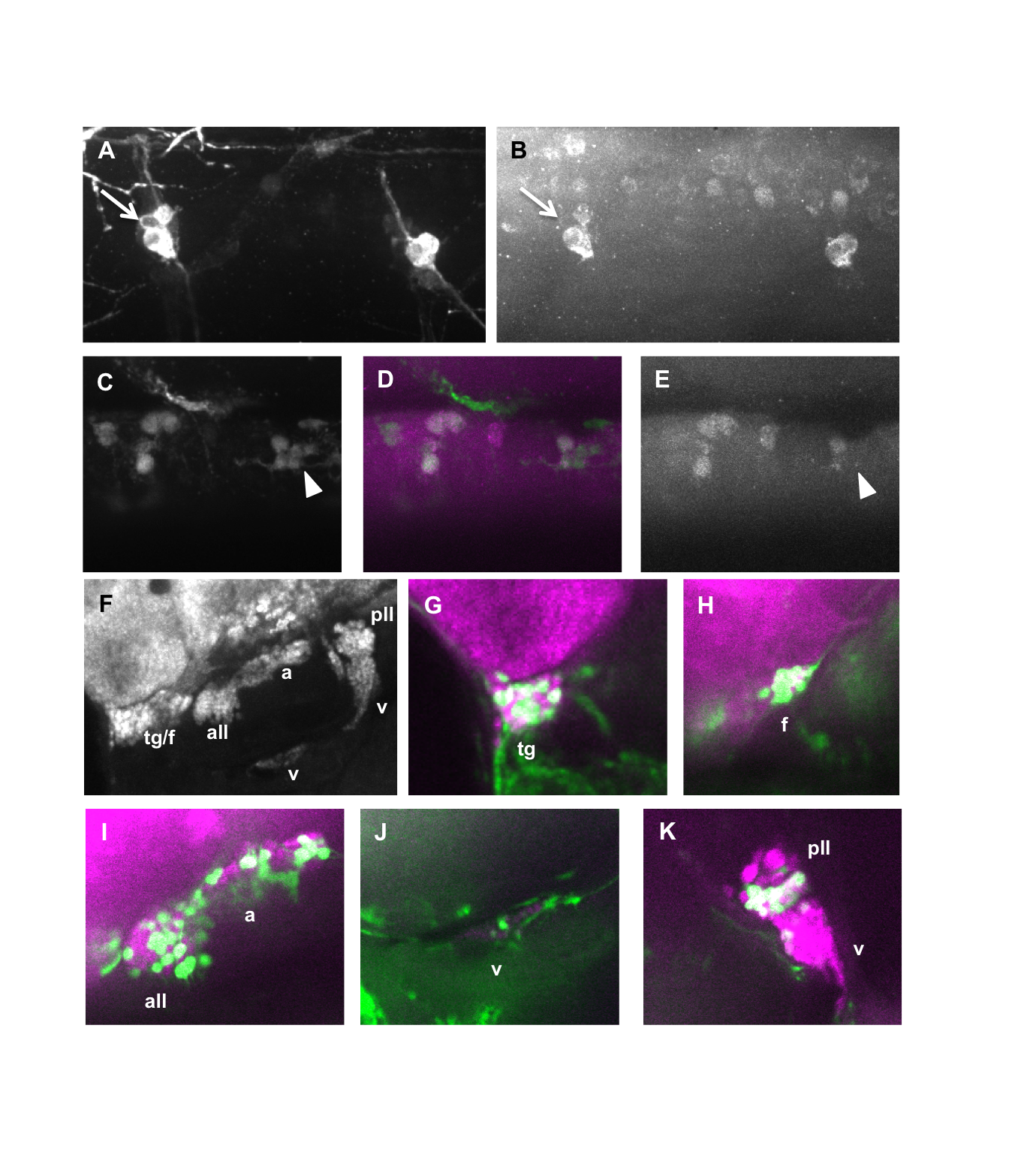

Supplement: Figure S1 — Cells of the peripheral nervous system are NC-derived. A, B) Combined GFP/HuC immunostaining reveals that neurons of the DRG are GFP+ (A) and HuC+ (B); there are also GFP+/HuC− cells visible in some ganglia (arrows), presumably Schwann cells. C–E) Enteric neurons are GFP+ (C) and HuC+ (E); in all panels with merged images (D, G–K), GFP is shown in green and HuC in magenta. There are also some GFP+/HuC− cells, which may represent NC-derived enteric glial cells (arrowheads). F) Antibody staining for HuC reveals neurons of the cranial sensory ganglia in a 4 dpf larva. In the trigeminal (G), facial (H), anterior lateral line (I), acoustic (I) and posterior lateral line (K) ganglia, there are numerous doubly positive neurons, indicating substantial NC contribution. In contrast, in the vagal ganglia, there are only a few GFP+ cells, which are not HuC+ (J, K). All images in D and G–K are single confocal slices. Abbreviations: a (acoustic ganglion); all (anterior lateral line ganglion); f (facial ganglion); pll (posterior lateral line ganglion); tg (trigeminal ganglion); v (vagal ganglia). (TIF) [file pone.0047394.s001.tif]
